# Evolution of Guidelines for Testosterone Replacement Therapy

**DOI:** 10.3390/jcm8030410

**Published:** 2019-03-25

**Authors:** Hyun Jun Park, Sun Tae Ahn, Du Geon Moon

**Affiliations:** 1 Department of Urology and Medical Research Institute of Pusan National University Hospital, Pusan National University School of Medicine, Busan 49241, Korea; joon501@naver.com; 2 Department of Urology, Korea University Guro Hospita, l No. 148, Gurodong-ro, Guro-gu, Seoul 08308, Korea; asturology@gmail.com

**Keywords:** testosterone, hypogonadism, men’s health, androgens

## Abstract

Testosterone is an essential hormone required for the developmental growth and maintenance of the male phenotype during the whole life. With the increasing male life expectancy worldwide and development of adequate testosterone preparations, the prescription of testosterone has increased tremendously. Testosterone replacement should be based on low serum testosterone and related clinical symptoms. In the last two decades, with the accumulation of data, official recommendations have evolved in terms of definition, diagnosis, treatment, and follow-up. In practice, it is better for physicians to follow the Institutional Official Recommendations or Clinical Practice Guideline for an adequate diagnosis and treatment of testosterone deficiency. Currently, four official recommendations are available for diagnosis and treatment of patients with testosterone deficiency. The inconsistencies in the guidelines merely create confusion among the physicians instead of providing clear information. Furthermore, there is no definite method to assess serum testosterone and clinical symptoms. In the era of active testosterone replacement therapy (TRT), physicians’ practice patterns should be consistent with the clinical practice guidelines to avoid the misuse of testosterone. In this review, the author introduces the evolution of clinical guidelines to provide a comprehensive understanding of the differences and controversies with respect to TRT.

## 1. Background

Testosterone is an essential hormone for males, required for differentiation, developmental growth, and maintenance of phenotype. Compared to the sudden decrease of estrogen with menopause in women, the level of testosterone declines gradually with aging in men. With men’s increasing life expectancy worldwide and development of proper testosterone preparations, the interest in and prescription of testosterone has rapidly increased [1].

Testosterone use in the United States tripled from 2001 to 2011, mostly in men without a clear reason [2,3]. In late 2013 and early 2014, two studies reported an increased risk of myocardial infarction and stroke associated with testosterone use [4,5]. After a decade of further research in this area, the percentage of men in the US receiving testosterone prescriptions decreased from 2013 to 2016. The steepest decline coincided with two published reports of testosterone-associated adverse cardiovascular events [4,5] and an FDA communication.

Besides the controversies regarding testosterone replacement therapy (TRT), the decision to recommend TRT should be based on low serum testosterone levels and related clinical symptoms. During TRT, efficacy and safety should be monitored. In practice, it is better for physicians to follow the Institutional Official Recommendations or Clinical Practice Guideline for proper diagnosis and treatment of testosterone deficiency in an era of expanding knowledge.

Dr. Morales first suggested clinical practice guidelines for screening and monitoring male patients receiving testosterone supplementation therapy as a treatment for erectile dysfunction in 1996 [6]. It was not an official recommendation but an expert opinion. In the last 20 years, several guidelines for TRT have been released from academic societies related to testosterone. At present, four different official guidelines have been released from the International Society for the Study of the Aging Male (ISSAM) [7], Endocrine Society [8], International Society for Sexual Medicine (ISSM) [9], and American Urological Association (AUA) [10] and will be updated as necessary (Table 1). Despite the importance of testosterone in men’s health and several guidelines from different academic societies, each guideline varies due to the differences in their specialty and interest. The inconsistencies in the guidelines merely create confusion among physicians instead of providing clear information. Despite the debates and controversies, consistency in the guidelines is very important for physicians to follow a well-defined protocol. In the era of active TRT, physicians’ practice patterns should be consistent with important clinical practice guidelines to avoid the misuse of testosterone.

In this review, the author introduces the evolution of clinical guidelines with respect to TRT to provide a comprehensive understanding of the differences and controversies. Nevertheless, it must be remembered that recommendations can never replace clinical expertise [7]. Treatment decisions, selection of treatment protocols, or choice of products for individual patients must take into account patients’ personal needs and wishes.

## 2. Nomenclature and Definition

For the last 60 years, the nomenclature of deficiency in testosterone production has changed several times [11,12,13,14]. The terminology of (partial) androgen deficiency (or decline) in the aging male has been advocated instead of andropause, which does not properly reflect the true clinical and biological picture. Previously, male menopause, known as androgen deficiency in the aging male (ADAM) and partial androgen deficiency in the aging male (PADAM) as a biochemical syndrome, was diagnosed by the decrease of testosterone levels. In 2002, the ISSAM suggested late-onset hypogonadism (LOH) or male climacteric syndrome as a clinical and biochemical syndrome associated with advancing age [15]. The ISSAM first released an Official Recommendation and updated the provisional recommendation in conjunction with the International Society of Andrology (ISA) and European Association of Urology in 2005 [16].

In 2006, Dr. Morales suggested testosterone deficiency syndrome (TDS) as the correct term instead of androgen deficiency or LOH as suggested by the Endocrine Society and ISSAM. According to the definition of LOH, it is exclusively associated with advanced age but ignores the fact that it can be detected as early as in the 3rd decade [14]. Furthermore, it is not uncommonly absent in the elderly. Androgen deficiency defined by the Endocrine Society refers to deficiency syndromes but mentions androgens in general instead of referring to testosterone (T) specifically. This is a problem because there is insufficient evidence to include other androgens such as dehydroepiandrosterone (DHEA) or androstenedione, while dihydrotetosterone (DHT) is generated locally in target tissues. In 2006, the Endocrine Society released the clinical practice guideline for testosterone therapy in adult men with evidence-based androgen deficiency syndrome [17] and updated it in 2010 [18]. The ISSAM published an evidence-based recommendation in multiple journals simultaneously to ensure a broad outreach to multidisciplinary audiences in 2008 [19] and updated it in 2015 [7]. Testosterone deficiency (TD) is newly used in the guidelines of 2015 by ISSAM [7], ISSM (International Consultation for Sexual Medicine, ICSM 2015) [9], and AUA (2018) [10]. In the 2018 recommendation, the Endocrine Society changed androgen deficiency syndrome to hypogonadism (Table 2) [8].

Despite the last 20 years of research on testosterone, the safety of TRT is still controversial. Due to the uncertainty regarding its safety, the Endocrine Society newly released an evidence-based guideline including level of evidence and grade of recommendation, which defines androgen deficiency syndrome only in men with symptoms and signs consistent with testosterone deficiency and unequivocally low serum testosterone levels. The ISSAM conducts research with aging males and its recommendations are described in view of TRT in the aging male.

In terms of the evolution of definitions, biochemical syndrome with low testosterone was changed to clinical and biochemical syndrome associated with advancing age in the ISSAM guideline; the ISSAM introduced LOH to help discriminate it from primary hypogonadism. LOH is diagnosed with typical symptoms and low testosterone, but hypogonadism or TD in adult men adversely affects multiple organ functions and quality of life. Among the various symptoms of testosterone action, decline in sexual function is the most closely associated symptom. Hence, the ISSM released the guidelines in the ICSM 2015 because of the importance of male sexual function. The AUA also released guidelines to emphasize the role of urologists and the importance of urological diseases in aging men, especially regarding sexual function and prostate safety.

## 3. Clinical Diagnosis Questionnaires

For the clinical diagnosis of LOH/TDS, several questionnaires have been developed [20,21,22] but the most popular are ADAM by Morley [21] and aging male symptom (AMS) questionnaire by Heinemann [22]. The ISSAM suggested seven categories as primary characteristic symptoms in 2002 [21], with sexual symptoms being the most prominent. In 2008, the ISSAM stated that ADAM and AMS are not specific for the diagnosis of LOH [19]. Although Morley considered bioavailable testosterone in ADAM [21], it cannot be used to monitor the response of patients after TRT because ADAM does not have a scoring system. During the construction of AMS, serum testosterone was not measured but symptoms of aging were gathered in healthy men [22]. In addition, AMS has a poor correlation with serum testosterone and low specificity for the diagnosis of LOH/TDS.

The Endocrine Society classified the symptoms and signs suggestive of androgen deficiency. They recommend measuring testosterone in patients with more specific symptoms and signs of androgen deficiency and consider measuring testosterone in patients with less specific symptoms and signs of androgen deficiency. In 2015, the ISSAM also classified the symptoms into sexual symptoms, non-sexual symptoms, and signs of hypogonadism [7]. In contrast, the ISSM recommended classifying symptoms into sexual, physical, psychological, and cognitive symptoms in the ICSM 2015 [9]. The 2018 AUA guidelines recommend that clinicians identify patients with symptoms associated with low testosterone, and conduct targeted physical exams for signs of low testosterone and measure it in all patients who have a history of conditions with risk of low testosterone, even in the absence of symptoms or signs of TDS [10]. The evolution of clinical diagnosis and questionnaires is summarized and presented in Table 3.

Despite the evolution of clinical diagnosis and questionnaires, the classification of sexual and nonsexual symptoms in ADAM [21] is still used in the recent recommendations released by the ISSAM. On the other hand, sexual, psychological, and somatovegetative symptoms in AMS [22] are used in the ISSM recommendations in addition to cognitive symptoms. Furthermore, the Endocrine Society and the AUA recommend using suggestive symptoms and signs to measure testosterone.

The clinical diagnosis of testosterone deficiency is only made when patients have low total testosterone levels combined with symptoms and/or signs. There is no definite questionnaire to diagnose LOH/TDS. It is impossible to develop such questionnaires because of the differences in symptoms and testosterone levels between people and different regions [23]. The use of validated questionnaires is not currently recommended to either define which patients are candidates for testosterone therapy or monitor symptom response in patients undergoing testosterone therapy. Despite the controversy and lack of recommendations, ADAM [21] is still useful as a screening tool for LOH/TDS for its simplicity, and AMS [22] may be useful to assess the presence and severity of symptoms and to monitor the response to TRT until the development of a more appropriate questionnaire.

## 4. Laboratory Diagnosis

Testosterone in blood is mainly bound to serum proteins with only 2% of the hormone circulating as free testosterone. Sex hormone binding globulin (SHBG) accounts for 60% of testosterone binding, and 40% of the total testosterone is bound by albumin or other proteins. Bioavailable testosterone, referring to free and albumin-bound testosterone together, is thought to reflect an individual’s biologically active, circulating testosterone. Biochemical parameters commonly used to assess androgen deficiency include total testosterone (TT), free testosterone, calculated free testosterone, bioavailable testosterone (bT), and free androgen index [24]. Theoretically, bT is the most accurate; however, a standard method to measure bT has not been established. Practically, morning TT is the most widely accepted substitute parameter, but results have been found to be misleading when SHBG is elevated. The diagnosis of low testosterone should be made only after two TT measurements have been taken on separate occasions between 8 and 11 am due to the circadian rhythm of testosterone production by the testicles. Free T measurement is recommended when TT measurement is not diagnostic. In individuals with clinically suspected TD, SHBG levels should be assessed if TT is low to normal or borderline, especially in obese or older men [9]. Calculation of free testosterone based on serum levels of testosterone and SHBG may be used to avoid inaccuracies in assessing the individual degree of androgenicity in a cost-effective manner [25,26]. Since normal ranges vary significantly between laboratories depending on the methods used and/or the assay kits employed, it is better to measure testosterone in the same laboratory [27].

Besides the variation in T assays, the cutoff value for low testosterone is different between studies [28,29] and societies [7,8,9,10]. The Endocrine Society [8] and the AUA [10] recommend using a TT level below 300 ng/dL with repeated measurements of morning TT as a reasonable cutoff in support of the diagnosis of low testosterone, preferably using the same laboratory with the same method/instrumentation for measurements [1,27]. The ISSAM [7] and the ISSM [9] use the cutoff value of TT <12 nmol/L or 350 ng/dL; they widened the indication of TRT to TT <350 ng/dL or 12 nmol in 2008 however in 2015 they suggested that TRT may be reasonably offered to symptomatic patients with TT concentration higher that 12 nmol/L based on clinical judgement (Table 4).

Recently, other hormones are also being measured as per recommendations. In patients with low testosterone, serum luteinizing hormone should be measured to establish the etiology of testosterone deficiency and may be an important factor in determining if adjunctive tests should be ordered [10]. Serum prolactin levels should be measured in patients with low testosterone levels combined with low or low/normal LH levels to screen for hyperprolactinemia. Persistently elevated prolactin levels can indicate the presence of pituitary tumors, such as prolactinomas [30]. Men with TT levels <150 ng/dL in combination with a low or low/normal LH should undergo a pituitary MRI regardless of prolactin levels, as this may indicate non-secreting adenomas [31].

## 5. Forms of Testosterone Supplements and Methods of Treatment

Since the discovery of testosterone by Butenandt [32], methods of preparations of testosterone have evolved over 80 years. Currently available preparations are oral, buccal, transdermal, subcutaneous, and intramuscular injection, which differ in formulations, route of administration, dose and interval to be used (pharmacokinetics), and safety profiles [33,34,35,36].

The treating physician should have enough knowledge and adequate understanding of the advantages and drawbacks of each preparation. The patient should be given the opportunity to actively participate in the choice of T formulation. The goal of testosterone therapy is the normalization of total testosterone levels combined with improvement in symptoms or signs [10,18]. Currently available testosterone preparations avoid 17-alpha-alkylated androgens and oral methylated formulations due to hepatotoxicity [34]. For rapid discontinuation in case of adverse effects, short acting preparations are preferred in the initial treatment. Commercially manufactured testosterone products should be prescribed rather than compounded testosterone, when possible. Additional monitoring and dose adjustments need to be performed to assure appropriate therapeutic levels if compounded preparations are prescribed [30].

Periodic hematologic assessment is indicated (i) before treatment; (ii) 3, 6, 12 months after initiating TRT, and thereafter annually; and (iii) with dose adjustments or change of preparation. Prior to commencing testosterone therapy, all patients should undergo a baseline measurement of hemoglobin/hematocrit (Hct). If the Hct exceeds 50%, TRT should be withheld until the etiology is formally investigated. While on TRT, Hct over 54% warrants intervention, such as dose reduction or temporary discontinuation. While the incidence of polycythemia for one particular modality of testosterone compared to another cannot be determined, trials have indicated that injectable testosterone is associated with the greatest treatment-induced increases in hemoglobin/Hct [33].

The dosing schedule of testosterone should be adjusted to achieve a total testosterone level in the middle tertile of the normal physiologic range of 450–600 ng/dL. The ISSAM recommended avoiding the supraphysiological level in 2005 but changed the recommendations to avoid the sustained supraphysiological level in 2008.

Patients on topical gels, patches, and intranasal formulations should have their testosterone checked between 2 to 4 weeks after commencement of therapy. The monitoring time of testosterone level is different according to the preparations based on the pharmacokinetic dosing principle of measuring steady state and with the intent of achieving therapeutic levels between 450–600 ng/dL. Three to six months after commencement of treatment, cessation of TRT should be considered in patients who experience a normalization of total testosterone levels but fail to achieve symptom or sign improvement. There is no utility in continuing testosterone therapy in men who achieve target testosterone levels without symptom/sign improvement.

Topical testosterone preparations (e.g., gels, creams, liquids) have the potential to result in transference to others. Women and children are at the highest risk of adverse events, such as virilization, precocious puberty, and hyperandrogenism [37].

Before TRT, prostate specific antigen (PSA) levels should be measured in patients over 40 years to lower the risk of occult prostate cancer. For patients who have an elevated PSA at baseline, a second PSA test is recommended to rule out a spurious elevation. In patients who have two tests results indicating elevated PSA levels at baseline, raising suspicion for the presence of prostate cancer, a prostate biopsy with/without MRI should be considered before initiating testosterone therapy. In public health reports, 39.3% of new testosterone users did not undergo a serum PSA test before initiating TRT and 56.7% did not undergo this test during the 12 months after TRT [1]. In the past, physicians have been concerned about the overscreening of prostate cancer, which can result in excessive biopsies and unnecessary treatments [38]. Despite the limited efficacy of screening PSA [39,40], the AUA guidelines recommend that prostate cancer patients on TRT should be monitored for PSA levels [10].

## 6. Assessment of Treatment Outcome

Testosterone testing and prescriptions have nearly tripled in recent years; however, it is clear from clinical practice that there are many men using testosterone without a clear reason [1,21]. Some studies estimate that up to 25% of men who receive testosterone therapy do not have their testosterone tested prior to initiation of treatment. Of the men who are treated with testosterone, nearly half do not have their testosterone levels checked after therapy commences [1,22]. According to the guidelines, the patient should be evaluated at 3 to 6 months after treatment initiation and then annually to assess whether symptoms have responded to treatment and whether the patient is suffering from any adverse effects.

Although various symptoms together indicate LOH/TDS or hypogonadism, each target symptom or tissue has a specific time frame of expected response to androgen replacement treatment [7,31]. In addition, improvement in hypogonadal symptoms and signs occur at different times for different organ systems. TRT options should be based firstly on symptoms and secondly on hormone concentrations, which should be evaluated on a symptom-specific basis. In the event that patients do not experience symptomatic relief after reaching the specified target testosterone levels or remain testosterone deficient in the setting of symptom/sign improvement, testosterone therapy should be discontinued. Patients should be informed that testosterone therapy may result in improvements in erectile function, low sex drive, anemia, bone mineral density, lean body mass, and/or depressive symptoms.

In 2008, the ISSAM described 3 to 6 months as a reasonable time interval for the improvement of libido, sexual and muscle function, and body fat, and 2 years for the improvement of bone density. Data regarding the time of the start of improvements and stabilization of each symptom were added to the accumulated data in 2015. In trials, patients with low testosterone have demonstrated statistically significant improvements in erectile function [41], sex drive [41], anemia [42], bone mineral density [43], lean body mass [44], and depressive symptoms [41]. However, given the limitations of the underlying studies and difficulties in assessing symptoms, it is unclear how clinically meaningful these improvements may be in some cases.

The evidence is less conclusive as to whether or not testosterone therapy improves cognitive function [45], diabetes [46], energy [47], fatigue [47], lipid profiles [46], and quality of life measures [48]. Despite the absence of definitive evidence, the panel of the 2018 AUA guideline suggests that patients with these symptoms may be counseled regarding the possibility of improvement following testosterone therapy [10]. With the increased male life expectancy and expansion of testosterone prescription, TRT use has shifted from testosterone-specific sexual symptoms to nonspecific generalized symptoms such as fatigue, poor concentration and depressed mood. To avoid misuse or abuse of testosterone, TRT should be done with patients’ consent after full explanation of the benefits and adverse effects of TRT.

## 7. Safety Concerns of TRT; Fertility

Compared with previous guidelines, concerns about fertility have been stated in recent guidelines [9,10]. Young hypogonadal men who need TRT should be informed about the concerns regarding fertility [49]. Before TRT, hypogonadal men should undergo testicular examinations to evaluate testicular size, consistency, and descent, and have hormonal status evaluation to assess their basal reproductive health status [10,41]. Exogenous testosterone therapy has been shown to interrupt normal spermatogenesis and can cause severely oligospermic or azoospermic states, and should not be used in men trying to conceive [50]. Instead of exogenous T, alternative therapies, including selective estrogen receptor modulators (SERMs) [51], human chorionic gonadotropin (hCG) [52], and aromatase inhibitors [53], are commonly used to promote the endogenous production of testosterone. Testosterone deficient patients with low or low/normal LH levels are also candidates for SERMs as a treatment for testosterone deficiency, particularly those wishing to preserve their fertility [33]. On the other hand, hCG is the only FDA approved alternative therapy for use in males. The long-term impact of exogenous testosterone on spermatogenesis should be discussed with patients who are interested in future fertility. Patients need to be made aware of the highly variable time course to recover sperm in the ejaculate and the variable degree to which the spermatogenesis function recovers after stopping exogenous testosterone [42]. The recovery of spermatogenesis after discontinuing the use of exogenous testosterone is also not well established in infertile males, and this important risk should be discussed with patients before starting treatment [43].

## 8. Safety Concerns of TRT; Cardiovascular Events

High body mass index coupled with low testosterone could put the patient at risk of cardiovascular events, and patients who are overweight or obese should be counseled regarding weight loss programs concurrent with testosterone therapy [54].

Low testosterone levels are associated with an increased incidence of major adverse cardiac events, such as myocardial infarction, stroke, and possible cardiovascular-related mortality, and an increased prevalence of certain atherosclerotic cardiovascular disease risk factors [44]. However, studies that measure cardiovascular benefit or harm in men on testosterone therapy have shown inconsistent and controversial results [44,55,56]. There is no definitive evidence linking testosterone therapy to a higher incidence of venous thromboembolic events [47]. All men with testosterone deficiency should be counseled regarding lifestyle modifications as a treatment strategy, such as losing weight or maintaining it within the recommended range, along with increasing physical activity, which has the potential to increase total testosterone levels and/or reduce signs and symptoms associated with testosterone deficiency.

## 9. Safety Concerns of TRT; Prostate

Among the various side effects of TRT, prostate safety is one of the major concerns. Hence, urologists are interested and play an important role in the diagnosis of testosterone deficiency and TRT. For the last 15 years, the prevalence of TRT has increased but its long-term risks are still controversial. The relationship between TRT and aggravation of lower urinary symptoms associate with benign prostatic hyperplasia, or development of prostate cancer has been debated. In recent randomized controlled trials, TRT did not significantly increase the rate of prostate cancer in testosterone deficient older men who received TRT compared with those who received placebo [42,45,46].

TRT in men with locally advanced or metastatic disease remains poorly understood and administration of testosterone in these scenarios should ideally be performed under research settings. TRT can be considered in completely cured patients after radical prostatectomy with favorable pathology (e.g., negative margins, negative seminal vesicles, negative lymph nodes) and undetectable PSA postoperatively [47].

Regarding TRT in men treated with radiation therapy (RT), it has been suggested that after RT, patients (with or without a history of androgen deprivation therapy) do not experience recurrence or progression of prostate cancer and experience either a steady decline in PSA values to <0.1 ng/mL or have non-significant changes in PSA [48].

There are limited data on active surveillance patients who are candidates for TRT. Available data indicate that patients with and without high-grade prostatic intraepithelial neoplasias who were on testosterone therapy did not experience significant increases in PSA or subsequent cancer diagnosis compared with men who did not receive testosterone [57].

## 10. Conclusions

Over the last two decades, various guidelines and recommendations for TRT have been developed and have evolved with different points of view, from expert opinions to official statements from the ISSAM, Endocrine Society, ISSM, and AUA. As medical knowledge expands and technology advances, guidelines will change with the accumulation of data. Despite the prevailing debates and controversies, consistency in guidelines is very important for physicians to follow a well-defined protocol in order to avoid the misuse of testosterone. Since official guidelines are updated with the accumulation of data and knowledge, physicians must be updated regarding these changes, and their practice patterns should be consistent with the most relevant clinical practice guidelines.

## Figures and Tables

**Table 1:** Evolution of guidelines for diagnosis and treatment of testosterone deficiency syndrome (TDS).

	Title	Year of Release and Update
Expert opinion	Clinical practice guidelines for screening and monitoring male patients receiving testosterone supplementation therapy.	1996
ISSAM	Investigation, treatment and monitoring of late-onset hypogonadism in males. Official recommendations of ISSAM.	2002, 2005, 2008, 2015
Endocrine Society	Testosterone therapy in men with hypogonadism.	2006, 2010, 2018
ISSM	Diagnosis and treatment of testosterone deficiency: Recommendations from the fourth international consultation for sexual medicine (ICSM 2015).	2015
AUA	Evaluation and management of testosterone deficiency.	2018

ISSAM: International Society for the Study of the Aging Male; ISSM: International Society for Sexual Medicine; ICSM: International Consultation for Sexual Medicine; AUA: American Urological Association.

**Table 2:** Evolution of nomenclature and definition.

	Title	Year of Release and Update
Expert opinion	Andropause, Male climacteric, Male menopause Androgen decline in the aging male (ADAM) Partial androgen decline in the aging male (PADAM)	Before official guideline
ISSAM	Late onset hypogonadism (LOH)	2002, 2005, 2008
ISSAM	Testosterone deficiency syndrome (TDS)	2008, 2015
ISSAM	Hypogonadism or TDS in adult men	2015
ISSM	Testosterone deficiency syndrome (TDS)	2015
AUA	Testosterone deficiency syndrome (TDS)	2018
Endocrine Society	Androgen deficiency syndrome	2006, 2010, 2018

ISSAM: International Society for the Study of the Aging Male; ISSM: International Society for Sexual Medicine; AUA: American Urological Association.

**Table 3:** Evolution of clinical diagnosis and questionnaires.

	Clinical Diagnosis & Questionnaires	Year of Release and Update
ISSAM	Saint Luis ADAM questionnaire Aging Male Symptom (AMS) Questionnaire; sexual, somatovegetative, psychological	2002, 2005
	AMS & ADAM are not specific	2008, 2015
	Sexual symptoms, nonsexual symptoms, & signs	2015
Endocrine Society	Measure testosterone in more specific symptom & sign Consider measuring testosterone in less specific symptoms & signs	2006, 2010, 2018
ISSM	Sexual, physical, psychological, cognitive symptoms	2015
AUA	Symptoms associated with low testosterone Signs associated with low testosterone	2018

ISSAM: International Society for the Study of the Aging Male; ADAM: androgen deficiency in aging male; ISSM: International Society for Sexual Medicine; AUA: American Urological Association.

**Table 4:** Cutoff values of testosterone for laboratory diagnosis.

	Cutoff Values	Year of Release and Update
Expert opinion	Total T (TT) or free T below the lower limits of normal	Before Official Guideline
ISSAM	TT < 231 ng/dL (8 nmol/L) TT: 231–346 ng/dL (8–12 nmol/L) or free T < 52 pg/mL	2005
	TT < 230 ng/dL (8 nmol/L) TT: 230–350 ng/dL (8–12 nmol/L) or free T < 52 pg/mL, SHBG	2008
	TT < 350 ng/dL (12 nmol/L) or free T < 65 pg/mL	2015
Endocrine Society	TT < 300 ng/dL or free T < 5 ng/dL	2006
	TT < 280–300 ng/dL or free T < 5–9 ng/dL	2010
	TT < 300 ng/dL or free T < 5 ng/dL	2018
ISSM	TT < 350 ng/dL (12 nmol/L)	ICSM 2015
AUA	TT < 300 ng/dL	2018

ISSAM: International Society for the Study of the Aging Male; ISSM: International Society for Sexual Medicine; ICSM: International Consultation for Sexual Medicine; AUA: American Urological Association.
